# A Joinpoint Regression Analysis of Syphilis and Gonorrhea Incidence in 15–19-Year Old Adolescents between 2005 and 2017: A Regional Study

**DOI:** 10.3390/ijerph17155385

**Published:** 2020-07-27

**Authors:** Anamaria Molnar, Mihaela Iancu, Rodica Radu, Cristina Maria Borzan

**Affiliations:** 1 National Institute of Public Health, Regional Center of Public Health Cluj, Louis Pasteur Str., no. 6, 400349 Cluj-Napoca, Romania; Anamaria.Brinzea@umfcluj.ro (A.M.); rodica.radu@insp.gov.ro (R.R.); 2 Department of Public Health, Iuliu Haţieganu University of Medicine and Pharmacy Cluj-Napoca, Avram Iancu Str., no. 31, 400083 Cluj-Napoca, Romania; borzancristina@yahoo.com; 3 Department of Medical Informatics and Biostatistics, Iuliu Haţieganu University of Medicine and Pharmacy Cluj-Napoca, Louis Pasteur Str., no. 6, 400349 Cluj-Napoca, Romania

**Keywords:** surveillance, syphilis, gonorrhea, adolescents, joinpoint regression

## Abstract

*Background and Objectives:* Surveillance of syphilis and gonorrhea in Romania is case-based and makes use of European case definitions. Adolescence is a period characterized by vulnerabilities and opportunities, a period when health decisions, including those related to sexually transmitted infections, may have a lifetime impact. The present study investigates the trends recorded in the incidence of syphilis and gonorrhea in 15–19 year-old adolescents in the central and northwestern regions of Romania. *Materials and Methods:* An observational study was conducted and this included surveillance data for syphilis and gonorrhea in the period 2005–2017 (*n* = 939). The distribution of demographic and epidemiological variables in adolescents with syphilis and gonorrhea was evaluated, as well as the tendency of the incidence of syphilis and gonorrhea in the studied population. Joinpoint regression analysis was used to characterize the incidence trend for syphilis and gonorrhea. *Results:* Between 2005 and 2017, 773 cases of syphilis and 166 cases of gonorrhea were reported. The incidence of syphilis and gonorrhea decreased. Most cases of syphilis have been found out by active detection. Most cases of gonorrhea have been found out by passive detection. The age distribution in the group diagnosed with syphilis was similar to that in the group diagnosed with gonorrhea. There was a higher frequency of syphilis in females and gonorrhea was more common in males. Syphilis was more common in rural areas. Gonorrhea was more common in urban areas. *Conclusions:* There was a decreasing tendency in the incidence of syphilis and gonorrhea in adolescents aged 15–19 during the studied period.

## 1. Introduction

Sexually transmitted infections are a major public health problem, both due to acute manifestations and long-term complications, with severe medical and psychological consequences for millions of people. In Romania, the surveillance of sexually transmitted infections is mandatory, comprehensive, passive, and based on the case [1,2].

The case definitions for the studied period are the same with the European case definitions, the last one being used in 2012 [3].

In Romania, the legislation for the compulsory declaration of sexually transmitted infections (syphilis and gonorrhea) dates from 1953 and it was further updated in 1971 [4], 2005 and 2014. [5,6].

Between 2005 and 2017, the trend of syphilis incidence increased in the EU/EEA countries, while in Romania, the trend of syphilis incidence decreased [7,8,9,10,11].

At the same time, between 2005 and 2017, in the EU/EEA countries the trend of gonorrhea incidence increased, while in Romania the trend of gonorrhea incidence decreased [7,8,12,13,14]. 

The World Health Organization (WHO) defines adolescents as those between the ages of 10 and 19 [15]. According to biological and behavioral characteristics, this period is further divided into two intervals - early adolescence (10–14 years old) and late adolescence (15–19 years old) [16].

Adolescence is an important period of formative growth and also a fascinating period of profound physical, psychological and emotional changes. It is a stage of life marked by both vulnerability and opportunity. The decisions adolescents make and the habits they gain can determine their health and well-being for a lifetime. Investments in adolescents’ health and their well-being are therefore highly important [17]. 

The 15–19-year-old adolescents (together with young adults aged 20–24 years) are at a higher risk of getting sexually transmitted infections than adults, due to a combination of biological, behavioral and cultural elements [18]. There is a continuous concern for promoting a healthy lifestyle and for reducing risk behaviors that also involve sexually transmitted infections during adolescence [19].

In European Union, there has been a continuous concern to study risk behaviors for contacting sexually transmitted infections in adolescents. Knowledge regarding sexually transmitted infections, proportion of condom users, number of sexual partners, history of sexually transmitted infections, early beginning of sexual relations, etc. were assessed [20,21,22,23,24].

Strategies that include evidence-based interventions for adolescent health have been developed in this respect. Thus, adolescents could benefit from all the rights and opportunities that a society can offer them, including the prevention and treatment of sexually transmitted infections [25,26,27,28]. 

The analysis of data regarding sexually transmitted infections obtained through surveillance or through various studies is the first step in implementing an adequate response to control these conditions [28,29,30]. 

The objectives of this study are to evaluate: (i) the relationships between the demographic and epidemiological characteristics in adolescents aged 15–19 years with syphilis and gonorrhea; (ii) the incidence trend for syphilis and gonorrhea in the studied population between 2005 and 2017.

## 2. Materials and Methods

A retrospective observational study was carried out for the period between 2005 and 2017.

The data were collected from eleven counties in central and northwestern Romania, with a total population of 4,832,078 in the last year of the research and a proportion of 15–19-year-old adolescents of 5.26% in the same year. Population data were updated every year and refers to population on 1st July for every studied year and geographic area and came from the National Institute for Statistics.

The data were collected by means of the sexually transmitted infections surveillance system [5,6].

All cases of laboratory confirmed syphilis and gonorrhea, carried out according to the regulations in force during the study period and reported in the age group of 15–19 years (*n* = 939), were included in the study.

Demographic and epidemiological variables were studied: age, gender, residence area, detection mode, case history of sexually transmitted infections (diagnosed sexually transmitted infections in the past) and, in the case of syphilis, the disease status (primary, secondary, early latent, late latent and unspecified latent classified according to European case definitions and Romanian legislation) [3,5,6].

The description of the qualitative characteristics in subjects with syphilis and gonorrhea was performed using absolute and relative frequencies (%), while the quantitative demographic characteristics were presented using the statistics of central tendency as the mean and standard deviation.

The testing of the differences in the frequency of syphilis and gonorrhea in relation to the demographic and epidemiological variables was performed by Student-*t*, Brown-Forsyth test, Chi-square or Fisher’s exact test.

The analysis of the trends in the incidence of syphilis and the incidence of gonorrhea was performed by joinpoint regression, a method that allowed us to test whether the incidence of syphilis and gonorrhea in the period 2005–2017 in the central and northwestern region of Romania is better explained by a single trend or by the existence of multiple trend segments (multiple trend patterns). Thus, joinpoint regression was used to identify those best-fitting break-points (years) in which there was a significant change in the incidence rate (crude incidence rates or unadjusted syphilis or gonorrhea incidence rate was defined as the total number of syphilis or gonorrhea cases divided by 15–19 old Adolescents population of the studied geography area and multiplied by a constant equal to 100,000) during the study period. For each trend segment, the annual percentage change (APC) was also estimated as a descriptive measure for describing changes in temporal trends. We also evaluated whether the estimated APC for each time segment was significant (APC other than 0) by calculating the associated 95% confidence interval.

The significance level chosen for all two-sided statistical tests was 0.05. Reported estimated significance levels (*p*-values) are not adjusted for multiple hypotheses testing. The software used for descriptive and inferential statistical analysis was the R platform (v3.6.1) (R Foundation for Statistical Computing, Vienna, Austria.). The joinpoint regression analysis was performed using the “segmented” R package. 

## 3. Results

### 3.1. Evaluation of the Frequency of Syphilis and Gonorrhea in Relation to Demographic and Epidemiological Variables

The study sample consisted of 939 adolescents and included 773 cases with syphilis and 166 cases with gonorrhea. The age distribution in the two groups was similar, the mean age in adolescents identified with syphilis being 17.6 ± 1.28 years, whereas adolescents with gonorrhea had a mean age equal to 17.66 ± 1.22 years (Student-*t* test, *p* = 0.975 > 0.05).

Gender distribution in the two groups was significantly different in the syphilis group compared to the gonorrhea group (Chi-square test, *p* < 0.001), with a higher frequency of syphilis among female adolescents (547, 70.8% vs. 226, 29.2%), while gonorrhea was more common in male adolescents (117, 70.5% vs. 49, 29.5%).

The adolescents’ residence area, was significantly associated with the type of diagnosis (Chi-square test, *p* < 0.001): syphilis is more common in adolescents from rural areas (420, 54.3% vs. 353, 45.7%) while gonorrhea was more common in urban areas (111, 66.9% vs. 55, 33.1%).

The highest number of cases of syphilis in adolescents was identified by active detection, while the highest frequency of gonorrhea was identified by passive detection (Table 1).

Among the adolescents with syphilis and gonorrhea, the most frequent cases were those who did not have a case history of sexually transmitted diseases, and, on the other hand, no statistically significant differences were found (Chi-square test, *p* = 0.466) on the frequency of case history of the sexually transmitted diseases in adolescents with syphilis vs. gonorrhea (3.6% vs. 4.8%). 

### 3.2. Temporal Distribution of Syphilis and Gonorrhea in Studied Population

The results showed that the frequency of syphilis was higher in girls than boys in each year of the studied period, while the frequency of gonorrhea was higher in boys (Table 2). 

### 3.3. Assessment of the Frequency of Primary, Secondary, Early Latent, Late Latent and Unspecified Latent Syphilis in Relation to Demographic and Epidemiological Variables

Syphilis cases were classified as primary syphilis (96, 12.4%), secondary syphilis (217, 28.1%), early latent syphilis (366, 47.3%), late latent syphilis (58, 7.5%) and unspecified latent syphilis (36, 4.7%) in the studied period and in the studied population, the most frequent being early latent syphilis.

The mean age in adolescents with primary, secondary, early latent, late latent and unspecified latent syphilis was significantly different (Brown-Forsythe test, *p* < 0.001), post hoc analysis showing significant age differences at the time of diagnosis in adolescents with secondary syphilis vs. early latent syphilis (17.2 ± 1.4 vs. 17.8 ± 1.2 years), and in adolescents with secondary vs. late latent syphilis (17.2 ± 1.4 vs. 17.9 ± 1.0 years).

The distribution of syphilis subtypes was significantly different in male and female adolescents (χ^2^ (4) = 80.55, *p* < 0.001), with primary syphilis being more common among male adolescents (64, 66.7% vs. 32, 33.3%), whereas secondary, early latent, late latent and unspecified latent syphilis were more common in female adolescents (secondary syphilis: 157, 72.4% vs. 60, 27.6%; recent latent syphilis: 277, 75.7% vs. 89, 24.3%; late latent: 49, 84.5% vs. 9, 15.5%; unspecified latent: 32, 88.9% vs. 4, 11.1%).

The frequency of primary, secondary, early latent, late latent and unspecified latent syphilis was similar in adolescents from urban and rural areas (χ^2^ (4) = 6.82, *p* = 0.146). We did not find a significant association between the presence of case history of sexually transmitted infections and the type of syphilis (Fisher’s Exact test, *p* = 0.498).

### 3.4. Syphilis and Gonorrhea Incidence Trends—Joinpoint Regression Analysis

Annual incidence of syphilis in 2005–2017 ranged from 37.3 to 3.9 per 100,000 adolescents; the negative linear trend over that time period was significant (*p* < 0.001). We found a significant change in incidence in the 2005–2017 period and two-trend periods were estimated for central and northwestern Romania with a joinpoint in 2011. The incidence rate decreased significantly by 97.9% per year for the 2005–2011 period, while for 2012–2017 there was a 64.7% decrease in the incidence rate, yet without statistical significance. 

**Figure 1:**
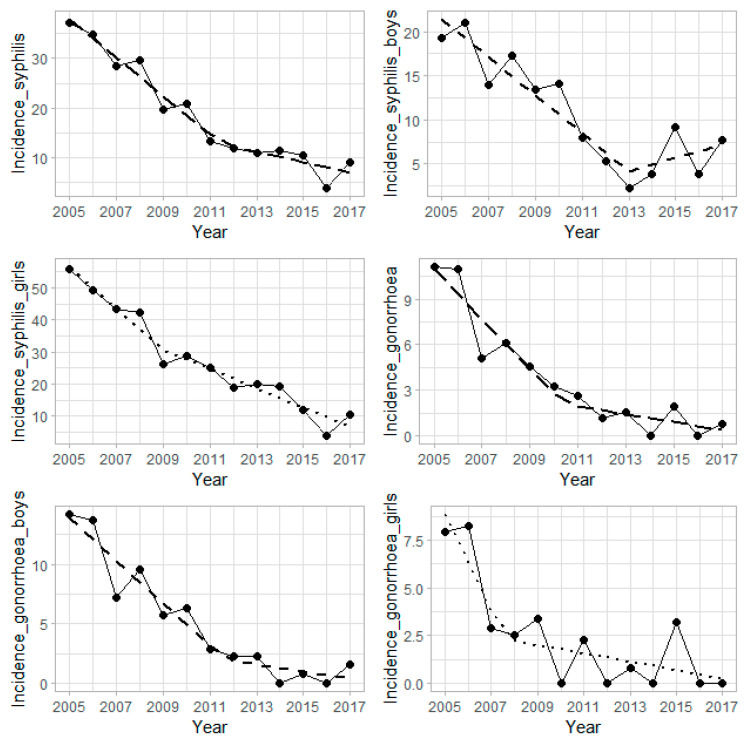
Trends in crude incidence rates of syphilis and gonorrhea in age groups of 15–19 years in the central and northwestern Romania, from 2005 to 2017. The analyzed temporal distribution was represented by continuous line graph, while estimated trends were marked by dotted lines.

For boys, the annual incidence of syphilis in 2005–2017 ranged from 21.1 to 2.3 per 100,000 cases; the negative linear trend over that time period was also significant (*p* = 0.0002). We found a significant change in incidence with a joinpoint in 2013 (Figure 1). 

The incidence rate decreased significantly by 88.4% per year for the 2005–2012 period, while for 2013–2017 there was an insignificant increase in the incidence rate, by 115.5%.

For girls, the annual incidence of syphilis in 2005–2017 ranged from 55.9 to 4.0 per 100,000 cases; the negative linear trend over that time period was also significant (*p* = 0.003). We found a significant change in incidence with a joinpoint in 2009. The incidence rate decreased significantly by 99.8% per year for the 2005–2009 period and 94.9% for the 2010–2017 period.

A significant change in the incidence of gonorrhea in 2005–2017 period with a joinpoint in 2010 was also found (Table 3). The significant change in trends of incidence rate was also found for boys and girls.

## 4. Discussion

This study aimed to assess the frequency distributions of syphilis and gonorrhea by demographic variables and to evaluate the longitudinal trends of these sexually transmitted diseases’ incidence using data reported from 2005 to 2017 in the central and northwestern regions of Romania.

### 4.1. Syphilis and Gonorrhea Frequency in Relation to Gender and Residence Area

We found that frequencies of syphilis infection were significantly different in adolescent boys and girls, with an observed higher frequency in women. Although our results are similar to those recorded at national level, as can be seen from the results of other studies [31], in Europe, different distributions of the frequency of syphilis can be observed in relation to gender, with an increased frequency among adolescent boys that may be partly due to sexual behavioral factors [32,33].

Our data demonstrated that a higher frequency of gonorrhea was noticed in urban areas and the results are consistent with previous studies that indicate that the frequency of sexually transmitted diseases is higher in urban areas where it is assumed that the population at risk is more concentrated [34].

### 4.2. The Trend of Syphilis Incidence Over the Studied Period

The overall trend of the incidence of syphilis in the EU/EEA countries between 2005 and 2017 was decreasing in the period 2005–2010 and increasing in the period 2010–2017, the tendency in the second interval was due to the increase in the incidence in males, given that the incidence of syphilis in female was decreasing [4,7,8,9,10,11].

In Romania, in the 2000–2010 period, rates decreased by 81% and in the 2006–2010 period, rates decreased by 68% [4]. However, they stayed still in 2009 with the highest rate (15.0 per 100,000 population) among countries with a comprehensive coverage reporting system [35]. The continuing decrease of syphilis rates included Romania in the group of countries with medium incidences in 2017, when the syphilis rate in EU/EEC was 7.1 cases per 100,000 population, while the incidence in Romania was 4.1 cases per 100,000 population [11].

In the European Union, in the age group of 15–19 years, the syphilis rate displayed minor fluctuations and stayed at low levels between 2008 and 2016; however, it increased by 19% in 2017, compared to 2016 [11]. Despite this, our results showed that, between 2005 and 2017, the incidence of syphilis in the central and northwestern regions of Romania in the age group of 15–19 years decreased, following the decreasing rates of this disease recorded in the population of Romania [4,11]. The main differences between European countries in incidence trends registered in adolescents regarding syphilis and gonorrhea could be due to the heterogeneity of surveillance systems (comprehensive vs. sentinel, compulsory vs. voluntary), the heterogeneity of case definitions and the heterogeneity of laboratory capabilities.

In the EU/EEA countries, between 2005–2013, the proportion of 15–19-year-old adolescents diagnosed with syphilis (out of all syphilis cases) registered a continuous decrease from 6.2% of the total syphilis cases in 2005 to 2.8% in 2013 [7]. In comparison, in the studied region, the proportion of 15–19-year-old adolescents diagnosed with syphilis (out of all syphilis cases) fluctuated between 12.37% and 9.3%, the values being higher than those registered in EU/EEA countries.

In 2015–2017 in Europe, in the age group of 15–19 years, rates of syphilis were higher in males than in females [9,10,11] relative to Romania where in 2013–2017, in the same age group, rates of syphilis were higher in females than in males [36,37,38,39,40]. In our study group, also the rates of syphilis were higher in females. In females, the incidence rates constantly decreased from 55.94 per 100,000 (15–19-year-old female population) in 2005 to 10.48 per 100,000 (15–19-year-old female population) in 2017. In males, the incidence rates constantly decreased from 19.31 per 100,000 (15–19-year-old male population) in 2005 to 7.68 per 100,000 (15–19-year-old male population) in 2017. Thus, the trends of syphilis incidence among gender groups indicated significant non-constant decrease over time, although the trend for adolescent boys showed increase without statistical significance from 2013–2017. A downward trend in syphilis incidence in the years 2005–2013 may have led to a decrease in the vigilance for prevention of syphilis which may have resulted in an apparent change in its trend for the period 2013–2017 in boys. The joinpoint analysis results showed that AAPCs were higher in girls than adolescent boys over the period 2005–2017.

In 2009, 2010, 2015–2017, for the entire EU/EEA, most cases were of early latent stage of syphilis, [4,9,10,11,35], although in 2017 some countries reported more than half of cases as primary or secondary stages of syphilis, reflecting the ongoing transmission and the need for public health interventions [11]. In 2016, 2017 Romania was in the country group which reported the most cases of early latent stage of syphilis; this situation may be the result of intensified screening [10,11]. In our studied group, also the most frequent diagnosis of syphilis was of early latent syphilis.

### 4.3. The Trend of Gonorrhea Incidence Over the Studied Period

The general trend of the incidence of gonorrhea in the EU/EEA countries between 2005 and 2017 had three ranges: a decrease period between 2005 and 2008, a slow increase period between 2008 and 2010, and a sharp increase period between 2010 and 2017. The increasing tendency in the second and the third interval was due to the increase in the incidence in males, given that the incidence of gonorrhea in female increased slowly [4,7,8,12,13,14].

In the 1990s Romania was part of the group of countries with very high rates of cases of gonorrhea per 100,000 population, yet also with rates which have decreased significantly since then (the same trend pattern also observed in the case syphilis). Romania was in the country group with more than 30% decrease rates of gonorrhea. Thus, in the 2000–2009 period, rates decreased by 53%, in the 2006–2009 period, rates decreased by 87% [35], in the 2000–2010 period, rates decreased by 90% and in the 2006–2010 period, rates decreased by 65% [4]. In 2009 and 2010, Romania was in the group of countries with the lowest incidence of gonorrhea in Europe: below 5.0 per 100,000 population. [4,35]. Romania was also in the group of countries with the lowest rates (≤1 per 100 000) in 2014–2017 period [12,13,14].

Between 2005 and 2013 in the EU/EEA countries the proportion of 15–19-year-old adolescents diagnosed with gonorrhea (out of all cases of gonorrhea) registered also a continuous decrease from 16.4% of the total cases of gonorrhea in 2005 to 11.8% in 2013 [7]. In comparison, in 2005–2017 period the incidence of gonorrhea in the central and northwestern regions of Romania in the age group of 15–19 years decreased, following the decreasing rates of this disease observed in Romania’s population [4,14].

Between 2005 and 2013, in the studied region, the proportion of 15–19-year-old adolescents diagnosed with gonorrhea fluctuated between 25.32% and 8.16%, and the values registered a more significant decrease compared to the decrease registered in the EU/EEA countries.

Between 2015 and 2017, in EU/EEA countries, the general population rates of gonorrhea were higher in males than in females: 32 vs. 8.8 per 100 000 in 2015, 30 vs. 9.5 per 100,000 in 2016, 35 vs. 11 per 100,000 in 2017 and for the age group of 15–19 years, the rates of gonorrhea were also higher in females than in males: 62 vs. 41 per 100,000 in 2015, 55 vs. 39 per 100,000 in 2016, 60 vs. 41 per 100,000 in 2017 [12,13,14].

Between 2013 and 2017, in Romania, for the age group of 15–19 years, the rates of gonorrhea were higher in males than in females [36,37,38,39,40]. In our study group, the rates of gonorrhea were also higher in males. In males, the incidence rates constantly decreased from 14.23 per 100,000 in 2005 to 1.54 per 100,000 in 2017. In females, the incidence rates also constantly decreased from 7.92 per 100,000 (15–19-year-old female population) in 2005 to no cases reported in 2016 and 2017. Our results are consistent with previous studies, which identified potential factors such as gender results that can have an influence on sexually transmitted diseases’ rates [41,42].

Both primary and secondary preventive strategies are known to reduce the risk for sexually transmitted infections [43]. It is important that preventive strategies be based on local epidemiological data [44]. There is a need for additional research regarding effective prevention strategies for sexually transmitted infections in adolescents [45].

Although, to our knowledge, there were not specific programs to enhance sexual health among adolescent population implemented during this period, prevention strategies for sexually transmitted infections were recommended.

Although the incidence for the two sexually transmitted infections is declining, they still represent a serious issue among adolescents. The dimension of the problem is not fully perceived because there are few studies which refer to the adolescent population. In this regard, ongoing surveillance efforts in this age group would be important. Ensuring access to medical services and proper treatment remains a priority, as it is also the case of prevention. Thus, there should be implemented information and education programs, especially before the start of sexual relationships, in order to reduce risky behaviors.

The results of the present study have to be viewed in the light of a retrospective, observational study design, the data collected came from the surveillance system of sexually transmitted infections. Based on the reported data, we analyzed the relationship between demographic variables (such as gender, age, residence area) and patterns of syphilis and gonorrhea incidence for the time period: 2005–2017. Therefore, taking under consideration of others potential sociodemographic factors such as sex education, lower cultural level, health education, education level in family that could explain the incidence trends of the two diseases is a limitation of the present study. This issue could be the subject of further/future research. Another perceived limitation of passive surveillance is under-reporting. This can lead to undetected trends. In addition, the results of this study should be generalized with caution in relation to the adolescents aged 15–19 years from Romania, mainly because further studies are needed to confirm the pattern of the incidence of syphilis and gonorrhea. 

Nonetheless, the present study is among the few studies in which the changes in growth and direction of syphilis and gonorrhea incidence rates has been analyzed in a population of adolescents.

## 5. Conclusions

The present study offers evidence for a decreasing trend of incidence rates for both syphilis and gonorrhea in 15–19-year-old adolescents in 11 counties in central and northwestern Romania, between 2005 and 2017. The joinpoint regression method highlighted changes in incidence trends from 2005 to 2017 for the two diseases by splitting time points of trends in two periods. Although there was a decreasing trend for the two diseases in the studied period, this decrease significantly slowed after 2012 for syphilis and 2010 for gonorrhea. In the study sample, the incidence rates of syphilis were higher in females and a non-constant decreasing trend for both males and females was highlighted. The similar results regarding differences in incidence trends by gender were noticed for gonorrhea incidence rate, further studies being necessary to explain the issues that underline these different trends.

A high proportion of cases of early latent, secondary and primary syphilis, seems to reflect ongoing transmission and the need for effective public health interventions. Public health interventions could include providing education and behavioral change, partner notification, treatment and screening for STIs in asymptomatic young adults, improving antimicrobial treatment compliance. 

## Figures and Tables

**Table 1:** Syphilis and gonorrhea frequencies in relation to demographic and epidemiological variables.

Variables	Syphilis Group (*n*_1_ = 773)	Gonorrhea Group (*n*_2_ = 166)
Gender, F_a_ (F_r_)		
Male	226 (29.2)	117 (70.5)
Female	547 (70.8)	49 (29.5)
The residence area, F_a_ (F_r_)		
Urban	353 (45.7)	111 (66.9)
Rural	420 (54.3)	55 (33.1)
Detection mode, F_a_ (F_r_)		
Active detection	584 (75.5)	65 (39.2)
Passive detection	189 (24. 5)	101 (60.8)
Sexually transmitted infections in case history, F_a_ (F_r_)		
No	745 (96.4)	158 (95.2)
Yes	28 (3.6)	8 (4.8)

F_a_ = Absolute frequencies expressed as number of cases; F_r_ = relative frequencies expressed as percentages.

**Table 2:** Number of syphilis and gonorrhea cases (%) by gender in the studied time period.

Year	2005	2006	2007	2008	2009	2010	2011	2012	2013	2014	2015	2016	2017
Syphilis													
Boys	38 (4.9)	40 (5.2)	25 (3.2)	29 (3.8)	21 (2.7)	20 (2.6)	11 (1.4)	7 (0.9)	3 (0.4)	5 (0.6)	12 (1.6)	5 (0.6)	10 (1.3)
Girls	106 (13.7)	90 (11.6)	75 (9.7)	68 (8.8)	39 (5.0)	38 (4.9)	25 (3.2)	24 (3.1)	25 (3.2)	24 (3.1)	15 (1.9)	5 (0.6)	13 (1.7)
All	144 (18.6)	130 (16.8)	100 (12.9)	97 (12.5)	60 (7.8)	58 (7.5)	36 (4.7)	31 (4.7)	28 (3.6)	29 (3.8)	27 (3.5)	10 (1.3)	23 (3.0)
Gonorrhea													
Boys	28 (16.9)	26 (15.7)	13 (7.8)	16 (9.6)	9 (5.4)	9 (5.4)	4 (2.4)	3 (1.8)	3 (1.8)	0 (0.0)	4 (2.4)	0 (0.0)	2 (1.2)
Girls	15 (9.0)	15 (9.0)	5 (3.0)	4 (2.4)	5 (3.0)	0 (0.0)	3 (1.8)	0 (0.0)	1 (0.6)	0 (0.0)	1 (0.6)	0 (0.0)	0 (0.0)
All	43 (25.9)	41 (24.7)	18 (10.8)	20 (12.0)	14 (8.4)	9 (5.4)	7 (4.2)	3 (1.8)	4 (2.4)	0 (0.0)	5 (3.0)	0 (0.0)	2 (1.2)

% Were reported to the syphilis sample size (*n*_1_ = 773) or gonorrhea sample size (*n*_2_ = 166).

**Table 3:** Syphilis and gonorrhea incidence trends among adolescents, stratified also by gender.

	Trend 1		Trend 2		AAPC, 95% CI for AAPC
	Years	APC	Years	APC	
Syphilis					
All	2005–2012	−97.9% ^#^	2012–2017	−64.7%	−2.57% (−2.98%; −2.16%)
Girls	2005–2009	−99.8% ^#^	2009–2017	−94.9% ^#^	−4.14% (−4.76%; −3.52%)
Boys	2005–2013	−88.4% ^#^	2013–2017	+115.5%	−1.18% (−1.62%; −0.74%)
Gonorrhea					
All	2005–2010	−80.8% ^#^	2010–2017	−23.1%	−0.88% (−1.08%; −0.69%)
Girls	2005–2008	−91.8% ^#^	2008–2017	−19.5% ^#^	−0.72% (−0.98%; −0.45%)
Boys	2005–2012	−83.3% ^#^	2012–2017	−24.2%	−1.12% (−1.36%; −0.88%)

^#^ Indicates that there was a significant change in the trend (*p* < 0.05); APC = Annual Percentage Change; AAPC = Average Annual Percentage Change determined as a weighted average of the estimated APC for the segments of 2005–2017; 95% CI = Confidence Interval.
